# COHELLP: collaborative randomized controlled trial on corticosteroids in HELLP syndrome

**DOI:** 10.1186/1742-4755-10-28

**Published:** 2013-05-22

**Authors:** Leila Katz, Melania Amorim, João P Souza, Samira M Haddad, José G Cecatti

**Affiliations:** 1 Obstetric Intensive Care Unit, Instituto de Medicina Integral Prof. Fernando Figueira, Recife, PE, Brazil; 2 Department of Obstetrics and Gynecology, Federal University of Campina Grande, Campina Grande, PB, Brazil; 3 UNDP / UNFPA / WHO / World Bank Special Programme of Research, World Health Organization, Geneva, Switzerland; 4 Development and Research Training in Human Reproduction, World Health Organization, Geneva, Switzerland; 5 Department of Reproductive Health and Research, World Health Organization, Geneva, Switzerland; 6 Department of Obstetrics and Gynecology, School of Medical Sciences, University of Campinas, Campinas, Brazil

**Keywords:** Severe preeclampsia, HELLP syndrome, Corticosteroids, Randomized controlled trial

## Abstract

**Background:**

Hemolysis, elevated liver enzymes, and low platelets (HELLP) syndrome is one of the most severe forms of preeclampsia and aggravates both maternal and perinatal prognosis. The systematic review available in Cochrane Library compared corticosteroid (dexamethasone, betamethasone, or prednisolone) given during pregnancy, just after delivery or in the postnatal period, or both before and after birth, with placebo or no treatment. Those receiving steroids showed significantly greater improvement in platelet counts which was greater for those receiving dexamethasone than those receiving betamethasone. There was no clear evidence of any effect of corticosteroids on substantive clinical outcomes. These benefits appear to be greater in Class I HELLP syndrome.

**Objectives:**

To determine the effectiveness of dexamethasone for accelerating postpartum recovery in patients with Class I HELLP syndrome in a multicenter randomized controlled trial.

**Methods/Design:**

The study is a triple blind randomized controlled trial including women with class I HELLP syndrome, and exclusion criteria were dexamethasone use in the last 15 days before diagnosis of HELLP syndrome; chronic use of corticosteroids; chronic diseases that alter laboratory parameters of HELLP Syndrome, such as chronic liver disease or purpura, patients unable to consent (coma or critical clinical condition) and without accompanying persons that may consent to study participation.

Eligible patients will be invited to participate and those who agree will be included in the study and receive placebo or dexamethasone according to a random list of numbers and subjects will receive the study medication every 12 hours for two days.

During the study the women will be subject to strict control of blood pressure and urine output. Laboratory tests will be performed at regular intervals during treatment and 24 hours and 48 hours after its suspension. If worsening of clinical or laboratory variables is observed, a rescue scheme of dexamethasone will be administrated. This proposal has already obtained approval of the local Institutional Review Board of the coordinating center (IMIP, Recife, Brazil), all other participating centers and of the National Council for Ethics in Research (CONEP) of the Brazilian Ministry of Health.

**Trial Registration:**

Clinical Trials Register under the number NCT00711841.

## Introduction

Hemolysis, elevated liver enzymes, and low platelets (HELLP) syndrome is one of the most severe forms of preeclampsia and aggravates both maternal and perinatal prognosis [1]. HELLP syndrome can be diagnosed during pregnancy or in the postpartum period and is associated with increased maternal risks, including liver hematoma, failure or rupture, pulmonary edema, renal failure, hemorrhagic complications and death. Perinatal prognosis is also poor due to preterm birth and growth restriction [2-4].

As a variant of severe preeclampsia, the only definitive treatment for HELLP syndrome is delivery of the baby and removal of the chorionic villi [4]. No specific treatment is available because the exact physiopathology of the disease remains unknown [5-7]. For this reason, treatment has historically been limited to controlling blood pressure, prophylaxis against convulsions, and termination of pregnancy [8].

Corticosteroids have been used for treating women with HELLP syndrome both before and after delivery. The suggested mechanism of action is a reduction in platelet adhesion, reduction in spleen platelet removal, a direct endothelial effect or rheological mechanism, and finally an increase in platelet activation [2].

Initial observational studies demonstrated improvement of maternal outcomes with administration of dexamethasone or betamethasone and small randomized clinical trials found improvement in some laboratorial tests, especially platelets count. The main outcomes, however, like death and maternal morbidities (liver hematoma, pulmonary edema, renal failure and *abruptio placenta*) were not affected by corticosteroid therapy [9-13].

The largest double-blind, placebo-controlled clinical trial carried out to date to evaluate this intervention was published in 2005. There was no difference between the two groups with respect to duration of hospitalization, recovery time for laboratory or clinical parameters, complications, or need for blood transfusion. These results remained unchanged, even following analysis stratified according to whether the patients were still pregnant or in the postpartum [14]. Notwithstanding, a trend for better maternal prognosis was found in most critically ill patients, diagnosed with class I HELLP Syndrome (platelets below 50.000/mm^3^). These patients had faster recovery of platelets and shorter duration of hospitalization.

A study carried out in a tertiary center in Brazil in 2008, failed to confirm any significant effect of the use of dexamethasone on the clinical and laboratory parameters of HELLP Syndrome in postpartum period. There was no difference in the evolution of laboratory or clinical parameters, frequency of maternal complications, need for rescue scheme, or length of hospital stay among patients who received dexamethasone and those who received placebo [15].

The systematic review available in the Cochrane Library identified 11 randomized controlled trials involving 550 women that compared corticosteroid (dexamethasone, betamethasone, or prednisolone) given during pregnancy, just after delivery or in the postnatal period, or both before and after birth, with placebo or no treatment. There was no clear evidence of any effect of corticosteroids on substantive clinical outcomes. Those receiving steroids showed significantly greater improvement in platelet counts which was greater for those receiving dexamethasone than those receiving betamethasone. The authors concluded that evidences are insufficient to demonstrate substantive improvement of clinical outcomes among women receiving steroids for management of HELLP syndrome. Notwithstanding, they suggest that the use of corticosteroids may be justified in clinical situations in which increased rate of recovery in platelet count is considered clinically worthwhile [16].

HELLP syndrome is a rare disease and its more severe forms are even rarer. To evaluate only the most severely ill patients, who are possibly those who would most benefit from corticosteroid therapy as shown in the study carried out by Fonseca et al. [14], various centers would have to be involved to achieve an adequate sample size with sufficient power to define infrequent outcomes such as death.

The overall objective of this study is to determine the effectiveness of dexamethasone to accelerate the postpartum recovery of patients with class I HELLP syndrome.

### Objectives and hypothesis

The overall objective is to determine the effectiveness of dexamethasone for accelerating postpartum recovery in patients with Class I HELLP syndrome in a multicenter randomized controlled trial.

#### Specific objectives

Analyzing patients with class I HELLP Syndrome randomized to receive dexamethasone or placebo, the specific objectives are to compare:

#### Primary outcome

•Frequency of composite maternal morbidity (acute pulmonary edema, eclampsia, bleeding manifestations, shock, coma, renal failure, liver failure, DIC, blood transfusion or death).

#### Secondary outcomes

•Laboratory parameters during and after treatment (hemoglobin, platelets, LDH, transaminases and bilirubin).

•Diuresis during and after treatment.

•Frequency of maternal complications: acute pulmonary edema, bleeding, acute renal failure and death.

•Need for rescue therapy regimen during the observation period.

•Time elapsed between the first dose of medication (dexamethasone or placebo) and egress (discharge or death).

#### Main hypothesis

In patients with class I HELLP Syndrome receiving dexamethasone, in comparison with placebo:

•The frequency of composite maternal morbidity is lower.

•Hemoglobin and platelets levels are greater while LDH, transaminases and bilirubin levels are lower.

•Better diuresis is achieved.

•Maternal complications are less frequent.

•There is less need for rescue therapy regimen during the observation period.

•Time elapsed between the first dose of medication and hospital discharge is lower.

## Methods/Design

### Study design

The present study is a triple blind randomized controlled trial.

### Study population and location

The study population will include all eligible women with Class I HELLP Syndrome hospitalized in the participating centers during the data collection period.

### Eligibility criteria

The inclusion criterion is the presence of Class I HELLP syndrome, both in antepartum or postpartum period. Exclusion criteria are: dexamethasone use in the last 15 days before diagnosis of HELLP syndrome; chronic use of corticosteroids; chronic diseases that alter laboratory parameters of HELLP Syndrome, such as chronic liver disease or purpura; patients unable to consent (coma or critical clinical condition) and without accompanying persons that may consent to study participation.

### Procedures for selecting participants and randomization

Eligible patients will be invited to participate and those who agree will be included in the study and receive placebo or dexamethasone according to a random list of numbers generated by the Random Allocation Software Ispharan Iran, version 1.0. This list of randomization will be provided by the statistician to the pharmacist who will be responsible for preparing the packages containing either the dexamethasone or placebo, both in an identical presentation, with the identification number of list labeled. This procedure will be followed in order to guarantee the concealment of allocation of patients in both arms. Considering dexamethasone has no important immediate identifiable effects, either patients and medical staff should be blind of the intervention condition in each case. Study medication and placebo, after delivered by the pharmaceutical industry to the pharmacy of the coordinating center, will be packed as previously described and according the random list. The pharmacy will be responsible for sending sets of 5 or 10 packages to each center depending on their performance on patients’ enrollment. The subjects will receive the study medication every 12 hours for two days.

During the observation period the women will be subject to strict control of blood pressure and urine output (diuresis evaluated spontaneous or urinary catheter). Laboratory tests will be performed at regular intervals of 24 hours (blood count, coagulation, renal and hepatic function) during treatment and 24 hours and 48 hours after its suspension. If investigators observe worsening of clinical or laboratory variables the study protocol will be interrupted and dexamethasone will be administrated. The same will be done if after the end of the two days the clinical situation deteriorates or does not improve. The patient will be considered as belonging to the group that she was originally randomized to, independent of later use of dexamethasone (type analysis intention to treat), recording such cases the need for "rescue therapy".

### Sample size calculation

The sample size for the trial was calculated using public domain software Openepi version 2.3.1. We assumed a rate of severe maternal morbidity condition of 50% among patients with syndrome HELLP in placebo group and 35% in patients treated with dexamethasone. With a power of 80% and an alpha error of 5%, 364 patients would be needed to demonstrate this difference. This number was increased to 400 patients for allowing for eventual losses and exclusions after randomization.

### Variables

#### Independent variable

use of corticosteroid (dexamethasone) or placebo.

#### Dependent variables

composite maternal morbidity, laboratory parameters during and after treatment (hemoglobin, platelet count, LDH, transaminases and bilirubin), diuresis during and after treatment, maternal complications, need for rescue therapy regimen, time elapsed between the first dose of medication (dexamethasone or placebo) and egress (discharge or death).

### Main outcomes

•Hypertensive disorders of pregnancy: defined according to the National High Blood Pressure Education Program Working Group on High Blood Pressure in Pregnancy [17].

•Maternal complications: any of these conditions: acute pulmonary edema, eclampsia, bleeding manifestations, shock, renal failure, coma, liver failure and blood transfusion.

•Composite maternal morbidity: presence of one or more of the following findings: acute pulmonary edema, eclampsia, bleeding manifestations, shock, coma, renal failure, liver failure, DIC, blood transfusion and death.

•HELLP syndrome: defined according to the criteria defined by Sibai [1]: 1. Hemolysis: characterized as the presence of abnormal peripheral blood smear (schystocytosis, anisocytosis, echinocytosis, poikilocytosis) associated with increased serum total bilirubin (> 1.2 mg%) or LDH increased (> 600 U/l); 2. Increased liver enzymes: when the serum AST is greater than 70 U/l or serum lactate dehydrogenase was higher than 600 U/l; 3. Thrombocytopenia: considered when the platelet count is less than 100.000/mm^3^.

•Rescue corticosteroid scheme: use of ampoules of intravenous dexamethasone in patients who had no improvement in laboratory tests or worsened in the course of the drug and therefore had the outline of the study (dexamethasone or placebo) suspended. Will also be adopted in patients who, after two days persisted with very altered laboratory tests.

### Data collection procedures

#### Data collection

Data will be inserted in an electronic clinical research form (CRF) especially developed for this study in an electronic platform (OpenClinica®) [18], which will be provided to the participating centers by the coordinating center. It was created by collaborators in CEMICAMP (Center for Studies in Reproductive Health of Campinas). After manual data collection in the questionnaire, it will be inserted in the electronic CRF in the project website and sent to the central database for storage. This was built in an exclusive computer, plus an external drive for backup, in an informatics unit with emergency power supply and automatic saving. Data missing in medical charts should be collected from other sources, such as hospital's database, prenatal cards, transference documents, etc. There will be a periodic communication between the coordinating center and the participating centers during the data collection period.

Every center will have a restricted area in the site, where they will have access only to their own cases through a password, with different levels of access depending on the hierarchical structure of the study in that site and the role played by personnel involved in the study. The principal investigators at the coordinating center and the administrator of the network will have global access to all cases. Summary information will be available in the form of monthly graphs and tables containing the number of cases included by each center and the distribution of referred diagnoses, provided by the coordinating center in the website's homepage.

In a monthly basis, the participating institutions will inform through the website the overall number of deliveries, live births and patients with HELLP syndrome (complete and partial) that occurred in the previous month and the total number of patients included. This data will be checked by the main local researcher at the end of each month. In order to minimize doubts from researcher assistants during data collection, a Manual of Operation will be developed with all information necessary to complete questionnaires and electronic CRF, use the internet, access to each center's database, standardized diagnostic definitions, among others.

A meeting will be held with all participating centers before the beginning of data collection, in order to standardize concepts and the data collection process with the completion of questionnaires. Training regarding the use of the electronic form will be performed by all centers, coordinated by the principal investigators, just before data collection is initiated, in order to standardize data collection. A final meeting will be held with the local principal investigators at the end of data collection process to discuss results, schedule final analyses, organize articles to be submitted for publication and define the responsibility and role of each person involved in this process.

#### Data analysis plan

**Figure 1:**
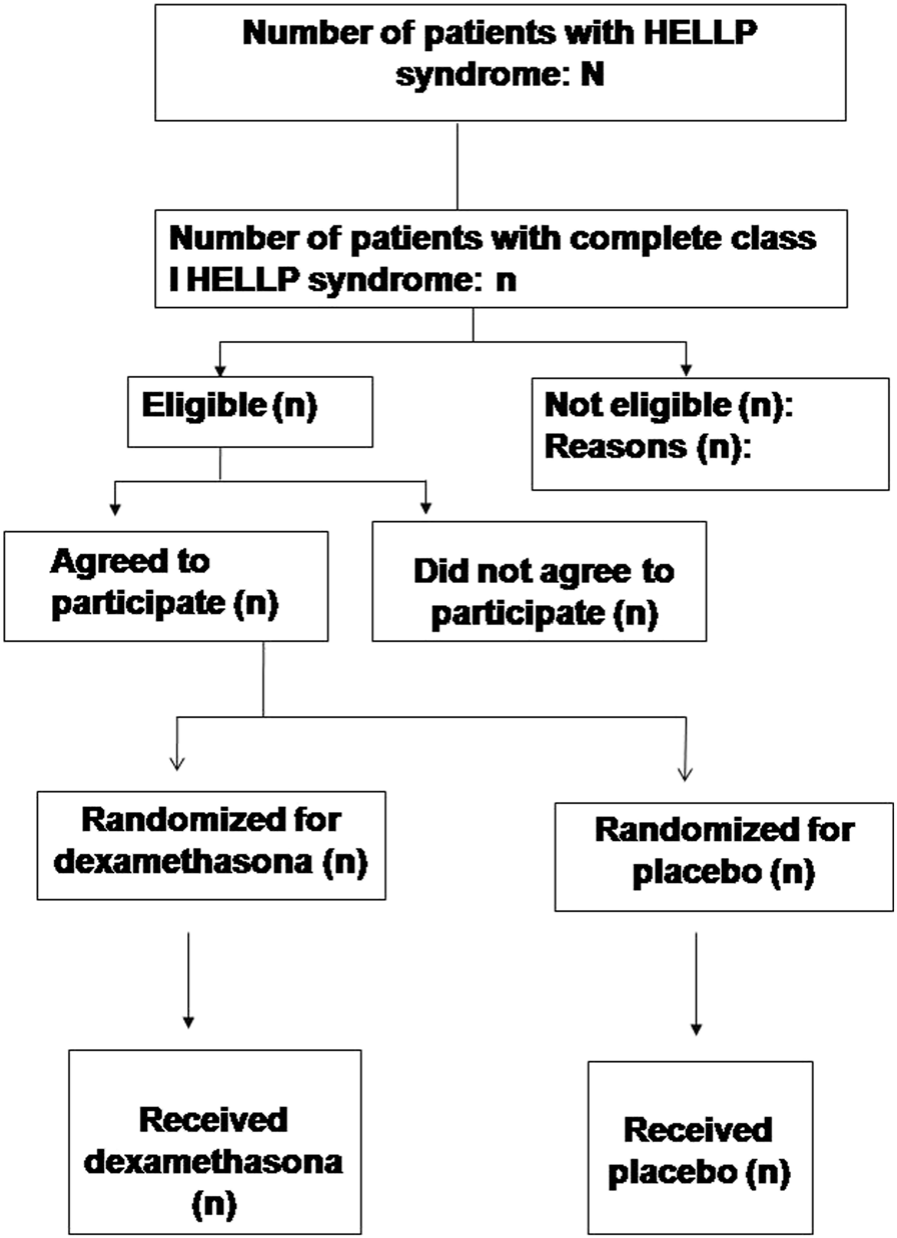
**Study design and population (CONSORT, 2010)**[19]**.**

The data analysis will be performed using the public domain software Epi Info version 7 (Centers for Disease Control and Prevention, Atlanta, GA), or the newest available version under the intention to treat principle. The statistician and the investigators will remain blind to the treatment groups until the tables will be prepared and the analysis concluded. The approach for analysis will be that showed in Figure 1 using an intention-to-treat strategy and following the correspondent recommendations from the CONSORT statement [19]. The characteristics of the participants in each group will be compared with Student’s *t* test for continuous variables with normal distribution and Mann–Whitney U test for discrete and ordinal variables or those with non-normal distribution. Categorical variables will be compared with Pearson’s χ^2^ test or Fisher’s exact test, as appropriate. P values for all tests will be two tailed at a 5% level of significance. Risk ratios and their 95% confidence intervals will be calculated as a measure of relative risk. The number needed to treat (NNT) and its 95% confidence interval will be calculated for the outcomes in which a beneficial effect of dexamethasone treatment is achieved, using the EBM calculator [ http://moosenose.com/EBCalculator.htm]; in case of adverse effects the number needed to harm (NNH) and its 95% confidence will also be calculated.

#### Quality control

The local researchers will maintain a record of problems occurred during the study and any doubt should be solved with the project's national coordinators and the Steering Committee.

### Study planning

In August of 2010, a meeting was held with the participating institutions in Recife, Pernambuco (northeast of Brazil) when representatives from 21 Brazilian health institutions were present. All these centers participated in the Brazilian Network for Studies on Reproductive and Perinatal Health [20] created in Campinas, Brazil, in 2008.

At this meeting, we presented the main points of the initial version of the proposal and all centers were invited to participate by filling out a form with the main characteristics of their institutions. Those centers that have met all requirements (at least 15 cases of HELLP syndrome treated per year with a minimum of five severe cases) were officially invited to participate and received a copy of the final draft, which was improved with substantial contributions from representatives of the participating centers.

#### Ethical issues

The original protocol of this research proposal has already obtained approval of the local Institutional Review Board from the coordinating center (IMIP, Recife, Brazil), from all other participating centers and of the National Committee for Ethics in Research (CONEP) of the Brazilian Ministry of Health, under the number 1255. The collaborating centers were only definitely incorporated in the study after the proposal has been approved by their respective Institutional Review Boards (IRB). The protocol also was published in the Clinical Trials Register under the number NCT00711841 (http://clinicaltrials.gov/ct2/show/NCT00711841). Patients with Class I HELLP syndrome will only be included if they agree to participate and sign the informed consent. In case of patients unable to consent (coma or critical clinical condition) the consent to participate will be given by the accompanying person. All principles related to research in human beings established by the Brazilian National Health Council according to the Declaration of Helsinki will be followed. The confidentiality on women's data and medical care will be ensured regardless of whether they participate in the study or not.

## Discussion

### Technical and scientific contributions of the study

Hypertension is the first cause of maternal death in Brazil and the third in the world. HELLP syndrome is one of the most severe forms of the disease and it is associated with several complications that include: hemorrhage, DIC, renal failure, liver failure and maternal death. These complications are strongly associated with Class I HELLP Syndrome and the possibility of accelerating recovery in these patients is very attractive with potential maternal benefits and substantial reduction in costs for health services. Although Class I HELLP Syndrome is relatively rare, we have a great number of patients with severe preeclampsia in the participant centers and a higher proportion of complicated cases because prenatal care is still inadequate and a lot of pregnant women are admitted with deteriorated clinical condition. As for instance, in the coordinating center, IMIP, the rate of HELLP Syndrome among patients with preeclampsia is so high as 16,2%. Furthermore, to determine if corticosteroids are indicated in this scenario will be relevant not only to Brazil but also to other countries with elevated frequency of hypertensive syndromes in pregnancy.

## Abbreviations

AST: Aspartate aminotransferase; CEMICAM: Center for Studies in Reproductive Health of Campinas; CRF: Clinical research form; DIC: Disseminated intravascular coagulation; HELLP: Hemolysis, elevated liver enzymes, and low platelets; IRB: Institutional Review Board; LDH: Lactate dehydrogenase; NNH: Number needed to harm; NNT: Number needed to treat.

## Competing interests

The authors declare that they have no competing interests.

## Authors’ contributions

The first version of this protocol was drafted by LK and MA. During the meeting held for the planning of the study implementation, investigators of all the participating centers contributed with suggestions. JGC revised the final complete version of the protocol. All authors have made substantive intellectual contributions to the manuscript and read and approved its final version.

## COHELLP Study Group

Luciana Pinheiro, Ivelyne R Araújo, Vera T Borges, Shirley K Bruno, Marcia P Bueno, Ricardo C Cavalli, Maria L Costa, Isabela Coutinho, Tábata Z Dias, Francisco E Feitosa, André V Florentino, Gilberto Gomes, Débora F Leite, Claudiney C Lotufo, Fátima A Lotufo, Nelson L Maia Filho, Coríntio Mariani Neto, Sérgio Martins-Costa, Elias F Melo Jr, Maria R Mesquita, Olímpio Moraes, Joaquim L Moreira, Daniela Nacarato, Bianca Nóbrega, Mary A Parpinelli, José C Peraçoli, Lúcia C Pfitscher, João L Pinto e Silva, Silvana M Quintana, José G Ramos, Marcos A Santos, Nelson Sass, Luiza E Schmaltz, Ricardo P Tedesco, Douglas B Tiago.

## References

[B1] Sibai BM The HELLP syndrome (hemolysis, elevated liver enzymes, and low platelets): much ado about nothing? Am J Obstet Gynecol 1990 162 311 316 10.1016/0002-9378(90)90376-I 2309811

[B2] Magann EF Bass D Chauhan SP Sullivan DL Martin RW Martin JN Jr Antepartum corticosteroids: disease stabilization in patients with the syndrome of hemolysis, elevated liver enzymes and low platelets (HELLP) Am J Obstet Gynecol 1994 171 1148 1153 10.1016/0002-9378(94)90054-X 7943088

[B3] Vigil-De Gracia PE Tenorio-Maroñón RF Cejudo-Carranza E Helguera-Martinez A García-Cáceres E Differences among preeclampsia, HELLP syndrome and eclampsia. Maternal Evaluation Ginecol Obstet Mex 1996 64 337 382 8925990

[B4] Maggann EF Martin JN Jr Twelve steps to optimal management of HELLP syndrome Clin Obstet Gynecol 1999 42 532 550 10.1097/00003081-199909000-00009 10451769

[B5] Thiagarajah S Bourgeois FJ Harbert GM Jr Caudle MR Thrombocytopenia in preeclampsia: associated abnormalities and management principles Am J Obstet Gynecol 1984 150 1 7 6476014 10.1016/s0002-9378(84)80100-3

[B6] Yalcin OT Sener T Hassa H Ozalp S Okur A Effects of postpartum corticosteroids in patients with HELLP syndrome Int J Gynecol Obstet 1998 61 141 148 10.1016/S0020-7292(98)00036-8 9639218

[B7] Suresh MS HELLP syndrome: An anesthesiologist's perspective Anesth Clin North Am 1998 16 332 348

[B8] O´Brien JM Shumate SA Satchwell SL Milligan DA Barton JR Maternal benefit of corticosteroids therapy in patients with HELLP (hemolysis, elevated liver enzymes and low platelets count) syndrome: Impact on the rate of regional anesthesia Am J Obstet Gynecol 2002 186 475 479 10.1067/mob.2002.121074 11904610

[B9] Heyborne KD Burke MS Porreco RP Prolongation of premature gestation in women with hemolysis, elevated liver enzymes and low platelets. A report of five cases J Reprod Med 1990 35 53 57 2299613

[B10] Magann EF Washburne JF Sullivan CA Chauhan SP Morrison JC Martin JN Jr Corticosteroid-induced arrest of HELLP syndrome progression in a marginally-viable pregnancy Eur J Obstet Gynecol Reprod Biol 1995 59 217 219 10.1016/0028-2243(94)01995-J 7657019

[B11] Heller CS Elliott JP High-order multiple pregnancies complicated by HELLP syndrome. A report of four cases with corticosteroid therapy to prolong gestation J Reprod Med 1997 42 743 746 9408876

[B12] Dreyfus M Tissier I Ndocko MA Denoual I Baldauf JJ Ritter J Corticosteroid therapy for conservative management in marginally-viable pregnancy complicated by HELLP syndrome Eur J Obstet Gynecol Reprod Biol 1999 85 233 234 10.1016/S0301-2115(99)00022-6 10584642

[B13] Schlembach D Munz W Fischer T Effect of corticosteroids on HELLP syndrome: a case report J Perinat Med 2000 28 502 505 11155438 10.1515/JPM.2000.068

[B14] Fonseca JE Mendez F Catano C Arias F Dexamethasone treatment does not improve the outcome of women with HELLP syndrome: a double-blind, placebo-controlled, randomized clinical trial Am J Obstet Gynecol 2005 193 1591 1598 10.1016/j.ajog.2005.07.037 16260197

[B15] Katz L Amorim MM Figueiroa JN Silva JL Pe Postpartum dexamethasone for women with hemolysis, elevated liver enzymes, and low platelets (HELLP) syndrome: a double-blind, placebo-controlled, randomized clinical trial Am J Obstet Gynecol 2008 198 3 2.83.e1-8 10.1016/j.ajog.2007.10.797 18194800

[B16] Woudstra DM Chandra S Hofmeyr GJ Dowswell T Corticosteroids for HELLP (hemolysis, elevated liver enzymes, low platelets) syndrome in pregnancy. Cochrane Database of Systematic Reviews The Cochrane Library, Volume 12 2010 Art. No. CD008148 10.1002/14651858 PMC4171033 20824872

[B17] Report of the National High Blood Pressure Education Program Working Group on high blood pressure in pregnancy Am J Obstet Gynecol 2000 183 1 S1 S22 10.1067/mob.2000.107928 10920346

[B18] OpenClinica® Community Edition: Open source for clinical research. Version 3.1-Community. 2004–2011 Akaza Research LLC and collaboratorshttp://www.openclinica.org21893916

[B19] Schulz KF Altman DG Moher D CONSORT Group CONSORT 2010 Statement: updated guidelines for reporting parallel group randomised trials BMC Med 2010 8 18 10.1186/1741-7015-8-18 20334633 PMC2860339

[B20] Brazilian Network of Studies on Reproductive and Perinatal Healthhttp://www.caism.unicamp.br/index.php/using-joomla/extensions/components/content-component/article-categories/219-rede-brasileira-de-estudos-em-saude-reprodutiva-e-perinatal

